# Geothermal sweetspots identified in a volcanic lake integrating bathymetry and fluid chemistry

**DOI:** 10.1038/s41598-019-52638-z

**Published:** 2019-11-06

**Authors:** Maren Brehme, Ronny Giese, Lily Suherlina, Yustin Kamah

**Affiliations:** 1 Helmholtz Centre Potsdam, GFZ German Research Centre for Geosciences, Geoenergy, Telegrafenberg, 14473 Potsdam, Germany; 2 TU Delft, Department of Geoscience and Engineering, Stevinweg 1, 2628CN Delft, The Netherlands; 3 Upstream Technology Center Pertamina, Jl. MedanMerdeka, Timur no. 6 Jakarta, Indonesia

**Keywords:** Geology, Tectonics

## Abstract

We investigate fluid pathways beneath volcanic lakes using bathymetry and geochemical measurements to locate best-possible drilling sites. Highly permeable structures, such as faults, provide fluid channels that are the most suitable access points to the geothermal resource. Accurate mapping of these structures therefore guides the successful targeting of wells. Lakes, rivers or ocean, can hide surface footprints of these permeable structures, such as in our case beneath Lake Linau. High-resolution bathymetry identifies linear and conical discontinuities, which are linked to offshore tectonic structures as confirmed by surrounding outcrops and hot springs. Geochemical measurements document inflow of hot saline acidic water into the lake verifying bathymetry-located highly permeable structures. Integrating onshore well data, our bathymetry and chemical results locates an ideal drilling site into the geothermal reservoir beneath the western shore line of Lake Linau.

## Introduction

Where to drill? This is the principle question arising when searching for an efficient access to subsurface geo-energy resources. Fluids in geological media flow along highly permeable fluid pathways, which are the key target for geothermal energy. These flow pathways can be faults, fractures or pore-throats. From wellbore, micro-earthquake and core data we know that the flow pathways are spatially correlated permeability structures^1^. This is true across five orders of magnitude in scale length from cm to km^1^. Available techniques to locate these structures have to be improved because only ~40% among these geothermal production wells perform above the expected well capacity^2^.

In geothermal fields, subsurface structural setting is identified by fault traces, hot springs or gas emanations at surface. Fluid discharge is an indicator for highly permeable zones at depth. These zones can be classified based on seismic interpretation of fault related, intrusion related or pipe related systems^3^. Fluids migrate in these systems bypassing the pore-network and sealing layers. Hot springs occur in specific structural settings along the faults^4^: at fault terminations, fault overlaps, fault intersections and topographically visible fault traces. The increased permeability is located in the damage zones of these settings^5^ due to enhanced deformation and instable geometry^6^. In the Taupo Volcanic Zone, e.g., fluid flow is mainly associated with fault step-overs and their linking faults^7^. Nevertheless, permeability can even vary within a single fault zone. This is because fault zones consist of two parts: the fault core with lower (matrix) permeability and the damage zone with higher (fracture) permeability^8^.

Mapping tectonic structures and fluid discharge onshore has been done mainly by structural geological mapping^9–12^, remote sensing^13,14^ and/or geophysical measurements^15,16^. However, using these approaches for offshore structures is not practical due to limited access to sea floor. Here, bathymetry is the most practical approach worldwide. Mapping seafloor morphology and therefore identifying tectonic structures has been done using bathymetry for more than 50 years now^17^. In the oil and gas industry, bathymetry is a well-proven tool to also detect hydrocarbon seepage at the seafloor^18,19^. At open sea, large faults have been characterized using this approach, e.g. off Portugal^20^ and in the Marmara Sea^21^. Bathymetry allows mapping the underwater morphology^22,23^, specific faults^24,25^, caldera structures and mud volcanoes^26,27^ under water. Furthermore, this approach allows identifying tectonic structures with enhanced fluid discharge under lakes^28,29^. However, bathymetry has not been yet used for mapping of sea bottom fluid discharge from a geothermal reservoir.

Seepage structures are described as pockmarks in lakes or subsea. Pockmarks are near-circular craters or depressions of various size and depth^30,31^. Their size range between 1–700 m with a depth range of 1–45 m, being much wider than deep. Pockmarks are known to be associated with fluid discharge, like gas emanations and warm water outflow^30,31^. Their relation to biological, mineralogical or physicochemical characteristics has been studied at different locations on the earth^32^, e.g. in Lake Constance they represent collapse structures formed by blown out material^33^. Pockmarks also show increased temperatures due to discharge of warm fluids^34^. They can also be controlled by tectonic structures or sea bottom current^35^.

The study site we chose to test the idea of mapping sea bottom geothermal fluid discharge is at Lahendong, which hosts a producing geothermal field on the Indonesian island of Sulawesi. Previous studies and personal communications with local inhabitants show, that an acidic volcanic lake in the middle of the geothermal field masks faults and fumaroles^36,37^. Furthermore, the most productive wells in the field penetrate into a reservoir compartment beneath this lake. However, onshore outcrops or hot springs are not sufficient to efficiently characterize the reservoir beneath the lake, which makes it difficult to determine future drilling targets. To tackle this issue, we used bathymetry and geochemical measurements to identify and characterize permeability structures of the geothermal reservoir hidden beneath the lake.

We carried out a ten-day survey on the 600 × 800 m large Lake Linau in the Lahendong geothermal field. The bathymetry survey was carried out using a Lowrance Elite TI 7 from Navico with CHIRP sensor installed on a raft driven in circles towards the middle of the lake. Geochemical measurements were done at 52 locations in the lake using a CastAway-CTD logger from SonTek. Four selected profiles are presented in this paper.

Results from our study show deep holes at the lake bottom. They are linked to known faults from previous studies and newly discovered faults in this study. Lineaments link fault outcrops onshore, hot springs on fault intersections and the deep holes offshore. The deep holes are located at typical ‘fault intersections’^4^. Geochemical data along and across these faults identify inflow of hot saline acidic water into the deep holes. This leads us to the conclusion that fault compartments have an increased permeability at the deep hole locations. Combining bathymetric and geochemical data, we were able to distinguish between permeable and impermeable fault compartments and relate them to productivity of geothermal wells. Such information is indispensable for a deep understanding of the behavior of the geothermal system and therefore its sustainable usage.

## Available Knowledge About The Reservoir Before The 2018 Survey

**Figure 1:**
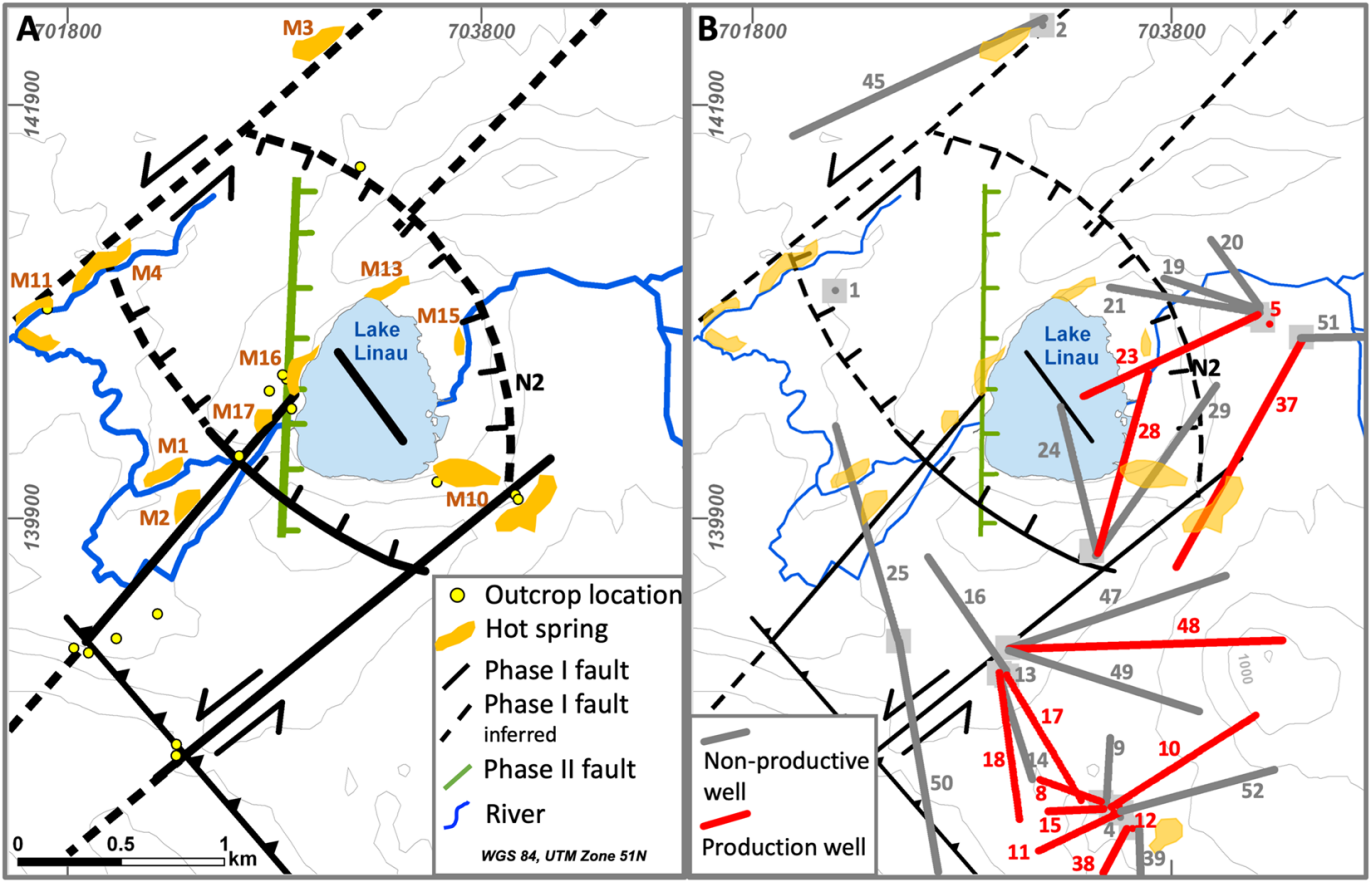
Fault set-up in the Lahendong geothermal field, outcrop and well locations before 2018.

The Lahendong geothermal field (LHD) has been producing steam and brine since 2001. The average permeability of the volcanic reservoir rocks is low with values from 10^−14^ to 10^−15^ m^2^. From 40 wells, 12 hit highly permeable zones and are sufficiently productive. The highest well productivities are from two wells targeting a reservoir beneath the caldera lake Linau. These wells (LHD 23 and LHD 28) produce 240 °C hot brine with a pH of 3 at a flow rate equivalent to 20 MW_e_. Despite the close proximity, other wells are considerably less productive (Table 1, Fig. 1). This inhomogeneity in production performance is due to a local control on the fluid flow by fractures and faults^36,37^. Previous geophysical studies, i.e. MT, microearthquakes and gravity, do not resolve fracture or fault locations beneath Lake Linau but rather show e.g. less resistive structures overlain by conductors, interpreted as clay cap above a regional fluid up-flow structure^38,39^. Therefore, this area lacks a full understanding of fault and permeability distribution especially beneath the lake.

**Table 1:** Approximate well productivities in the Lahendong geothermal field.

Well No	Productivity [MWe]	Well No	Productivity [MWe]	Well No	Productivity [MWe]
1	0	15	5	29	0
2	0	16	0	37	3
4	7	17	7	38	7
5	5	18	7	39	0
8	5	19	0	45	0
9	0	20	0	47	7
10	5	21	0	48	7
11	5	23	20	49	0
12	5	24	0	50	to be tested
13	0	25	0	51	to be tested
14	0	28	20

### A priori knowledge on faults before the 2018 survey

Location of faults in the Lahendong geothermal field have been investigated in a number of previous studies^36,37,40–42^. Most obvious indicators for faults are their appearance in outcrops, topographical steps in the field and well data on geochemistry, productivity and temperature. Hot springs often occur at intersections of faults, validating them as additional indicators for faults^37^.

The tropical environment of Lahendong with dense vegetation and high alteration rates allows only rare access to outcrops with fresh hard rock. However, data from 40 wells show indications for faults through strong differences in geochemical fluid properties, productivities and temperatures in close distance. Mainly two geochemical types of fluids are produced from wells, i.e. one highly saline (5–10 mS/cm) and one less saline (1 mS/cm). Wells producing highly saline fluids from below Lake Linau (LHD23, LHD28) are located only 300 m from a well producing low saline fluid (LHD5) (Fig. 1). An impermeable fault (N2) prevents mixing between those fluids at depth. Also, high productive wells and less or non-productive wells are in close distance but separated by faults: While LHD 23 produces 20 MW_e_, LHD 19 is non-productive similar to the non-productive LHD 24 close to LHD 28 producing 20 MW_e_ (Table 1, Fig. 1). Additionally, temperature data from wells show strong drops from 310 °C to 160 °C across the fault N2 from LHD 23 to LHD 5, indicating a cold intrusion through vertical permeability in the fault. Thus, faults in the Lahendong area can be horizontally impermeable but permeable in vertical direction independent of fault type.

A comparison of fault locations with hot springs in the Lahendong area show that hot springs occur at typical structural settings^4^ (Fig. 1), i.e. fault tips (M11), fault intersections (M4, M10, M16) and along fault traces (M2, M3). Hot springs, which do not show a clear link to faults are assumed to be sitting on hidden faults.

The combination of previous knowledge on fault location and type suggests two faulting phases in the Lahendong area (Fig. 1). The relatively older one (Phase I) is situated in a stress field with horizontal σ_1_~355°. In this system, strike-slip faults strike 40° and have a left lateral movement also according to outcrop observations, e.g. slickensides. Step-over zones are observed, creating thrust faults at a right step-over and normal faults at a left step-over. This stress field is generated by two subduction zones pushing below North-Sulawesi from north and south. A second faulting phase (Phase II) is caused by upwelling of the regional crust due to heating and nearby volcanism. This stress-field with a vertical σ_1_ creates N-S or E-W oriented normal faults. Normal faults in both systems dip 70–90°, while thrust faults dip with ~40° and strike-slip faults are nearly vertical.

In spite of this comprehensive knowledge, the geothermal exploitation area especially beneath Lake Linau is lacking a detailed understanding of fault orientations and their permeability behavior. The reservoir beneath Lake Linau is still a blank area but of special interest because of its high productivity and exceptional fluid composition (highly saline and highly acidic), which brings challenges for a sustainable production.

The results of the study we report here clarify most of the open questions related to the poorly understood variability in the productivity of geothermal wells and brings a detailed picture of the reservoir beneath Lake Linau including new target zones for wells.

## Methods

### Bathymetry

Bathymetry has been done using a Lowrance Elite TI 7 from Navico. The tool consists of a CHIRP (Compressed High Intensity Radar Pulse) sonar, GPS navigation and a multibeam echosounder with side-scan and down-scan imaging. The down-scan detects the depth at a single point and the side-scan captures information and views at each side of the boat.

**Figure 2:**
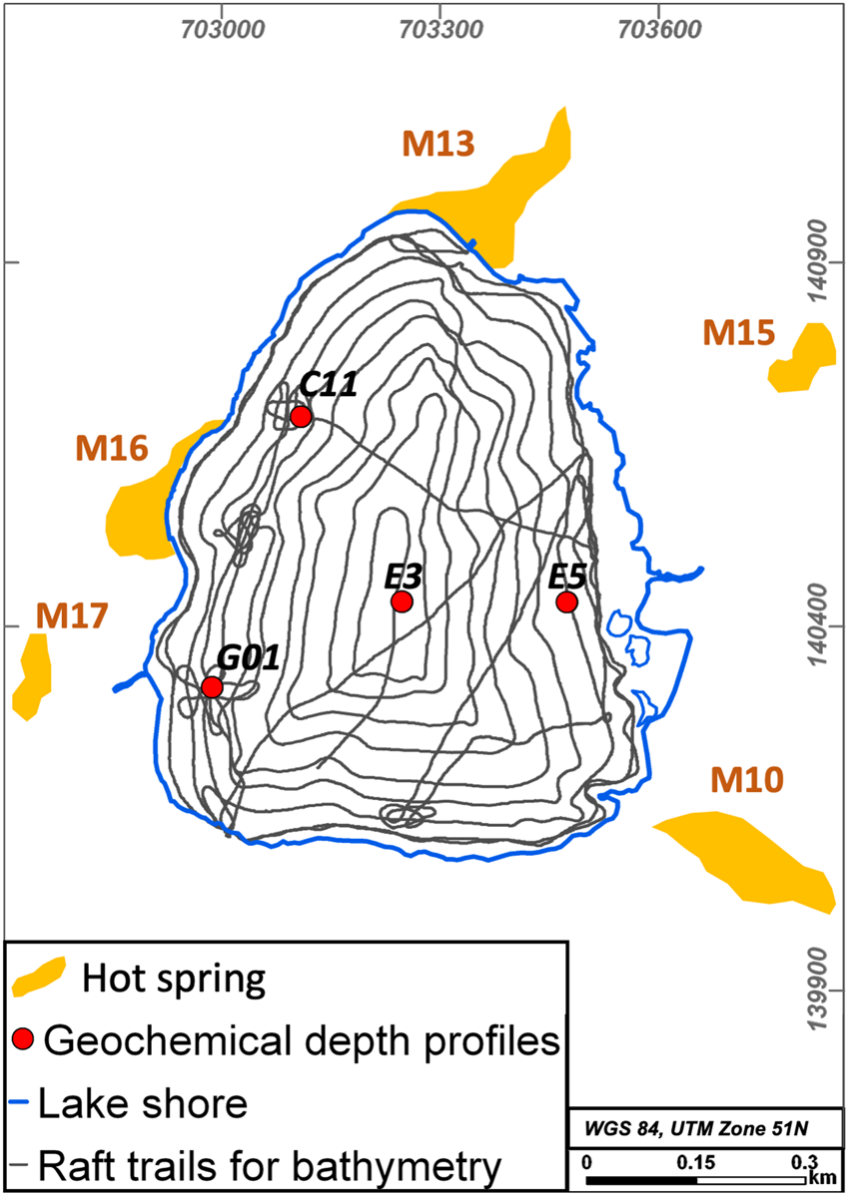
Measurement set-up for bathymetry driven by the raft and geochemical depth profiles at sampling locations G01, C11, E3, E5 and hot spring locations.

Circles driven on the lake have a distance of 40 m and add up to a total length of 16.5 km, which is equal to 15,000 measurement points (Fig. 2).

Instruments have been installed on a raft going in spirals on the lake with decreasing diameter to the lake center and a distance of approximately 40 m between the consecutive rounds. Certain areas were crossed with an additional finer mesh to get a more detailed data set.

Thereafter, data have been processed and transformed to x-y-z coordinates by Kriging analysis using ReefMaster by ReefMaster Software Ltd. Processed depth data achieve an accuracy of 0.5 m. 3D views of bathymetry data and fault locations have been achieved by self-written codes in Matlab.

### Geochemical depth profiles

Chemical profiles have been logged using the CastAway-CTD logger from SonTek. The logger includes sensors for conductivity, density, temperature, pressure and GPS. It allows sampling at a 5 Hz sampling rate up to 100 m depth, translated to 15 cm sampling distance.

52 spots were sampled across the lake at 100 m distance from a raft on the lake. Three to four logs were run consecutively at the same spot. Logging is done while lowering the probe to the lake bottom and lifting it again with approximately 1 m/s. Sampling spots presented in this paper are two deep holes in the west, one spot in the center of the lake and one spot at the eastern shore of the lake (Fig. 2). The selected spots represent two geochemically extreme pattern (G01, E3, E5) and one average pattern (E3) for this lake. Logs presented for one single spot show the most representative average pattern for that location. The whole dataset of 52 spots is topic of a subsequent manuscript in progress.

Data were extracted from the instrument using Castaway-CTD Software by SonTek. Plots of geochemical depth profiles were constructed from self-written codes in Matlab.

## Results

### Bathymetry

**Figure 3:**
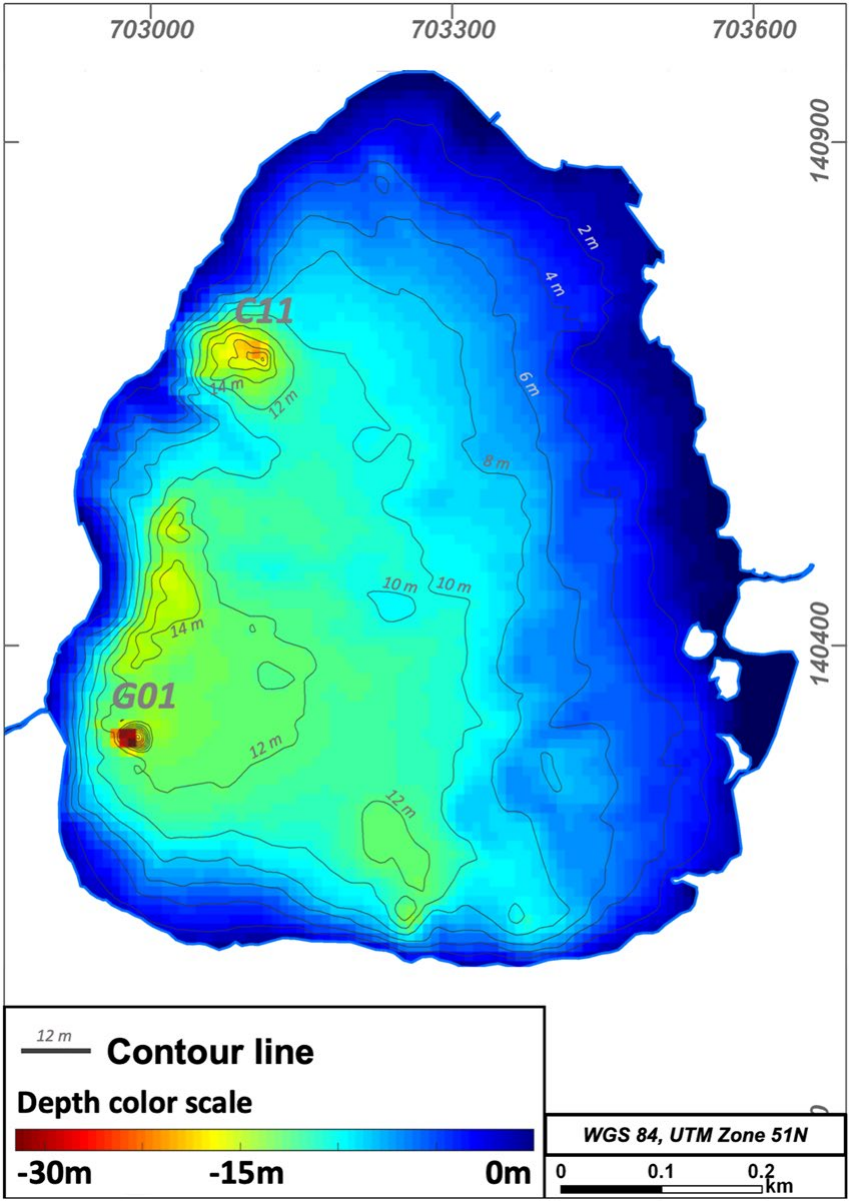
Bathymetry depth map of Lake Linau.

This is the first reported bathymetry survey for Lake Linau. Before, the depth of the lake was estimated to 10–12 m^43^. Its total depth ranges from 0.5 to 35 m. In general, the northern and eastern parts of the lake are shallower, with <10 m. Here, the lake bottom dips with ~2° towards the southwest. The southern and western parts dip with ~10° and have a maximum depth of 14 m (Fig. 3).

**Figure 4:**
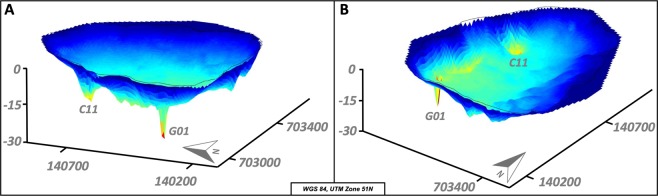
3D bathymetry of Lake Linau, (**A**): view from SW, (**B**): view from SE, vertical exaggeration ~10.

The main discovery of the survey were two 22 m and 35 m deep holes at the lake bottom near the western shore. The holes have a diameter of 20–40 m at the top and 4 m at the bottom, which indicates a slope of the conical walls of 34°. Side-scan images show that the holes are not filled by lake sediments and the walls are built up of the surrounding bedrock. In between the two deepest holes the lake bottom has several 14 m deep elliptical structures sitting on an alignment striking N-S. Further round-shaped depressions are 12 m deep in the southern part of the lake (Figs 3 and 4). We traversed the deeper structures several times with a denser net of raft trails to map them in more detail.

**Figure 5:**
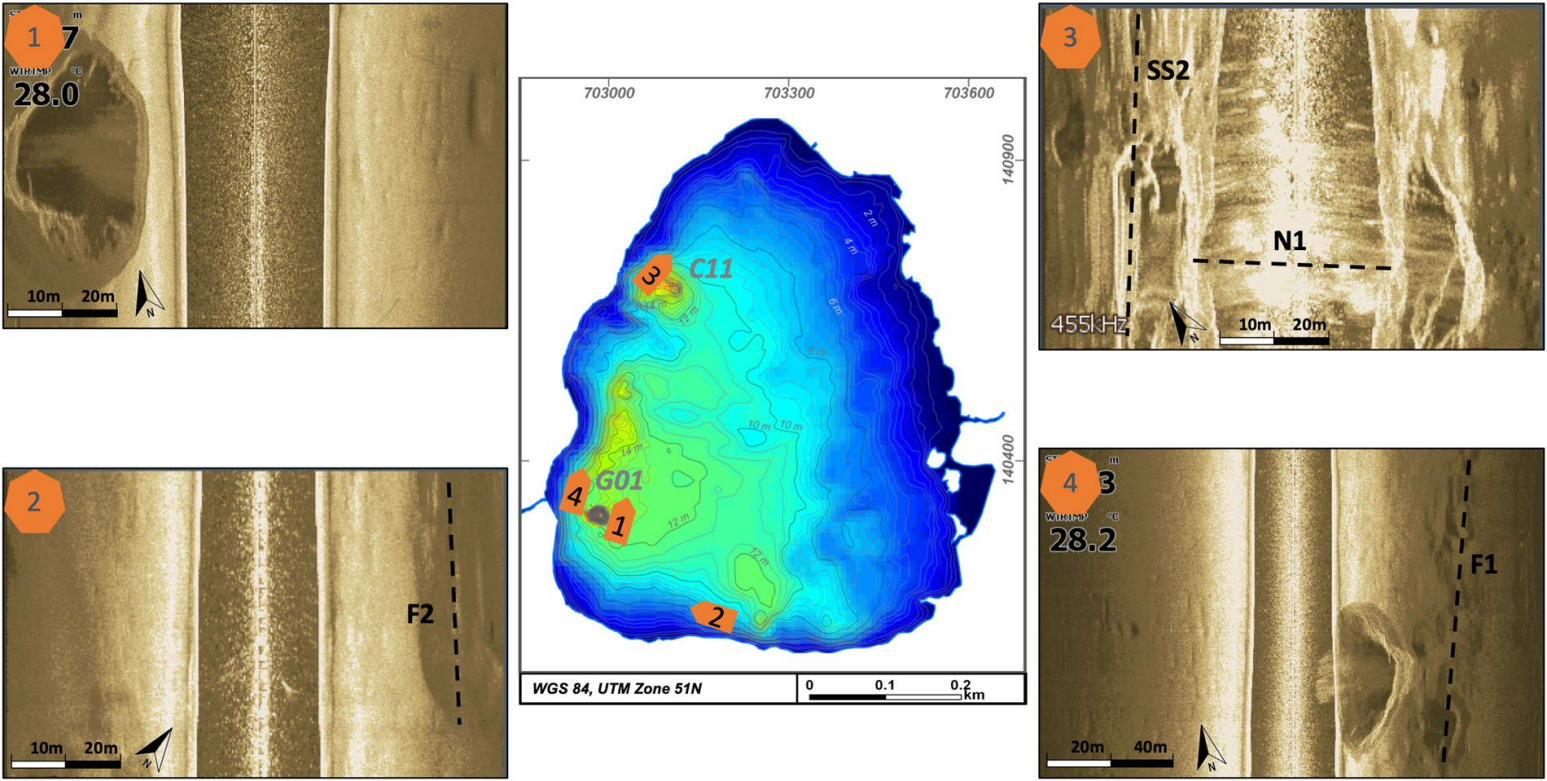
Side scan images from the lake bottom showing hole structures (**1**,**3**,**4**) and fault patterns (**2**).

**Figure 6:**
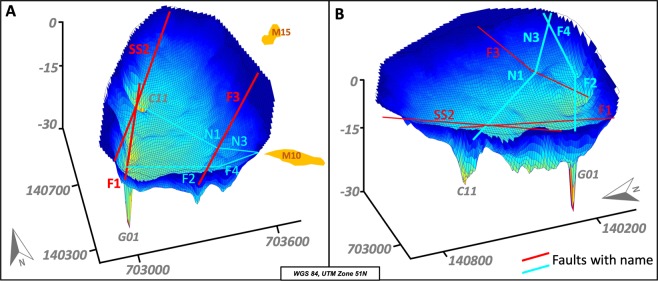
3D bathymetry with newly discovered faults and their names, vertical exaggeration ~20.

Side-scan images show the hole structures and lineaments at the lake bottom (Fig. 5). The deepest hole (locations 1 & 4) is imaged from east and west. The image shows the strongly dipping conical walls. At C11 the image is less clear but still shows the hole structure. The lake bottom here is covered by soft sediment (light ground color) transported in from hot spring discharge and surrounding alterations at M16. At location 2 a linear element is visible to the NE, which is assumed to be a fault structure. Also, at location 3 and 4 structures on the lake ground are visible related to fault lineaments shown in Fig. 6.

Especially the 3D presentation of the bathymetry data show lineaments at the lake bottom, assumed to be fault traces (Fig. 6). The two deepest holes and depressions in between sit on the most obvious lineament striking NNE-SSW (F1). In the deep hole C11, F1 meets with the strike-slip fault SS1 and the normal fault N1. In the eastern part, the lake bottom shows topographic break-offs and deeper round-shaped structures in alignment with hot spring M15 striking NE-SW (F3). G01 is the intersection point of two lineaments (F1, F2) striking NE-SW and NW-SE. F2 crosses depressions in the southern lake area and continues as F4 towards hot spring M10 onshore where it meets with N3. N3 is the connection line between the previously known fault N1 and M10 onshore. N3 and N1 meet at the break-off point in the eastern lake part.

### Geochemical depth profiles

**Figure 7:**
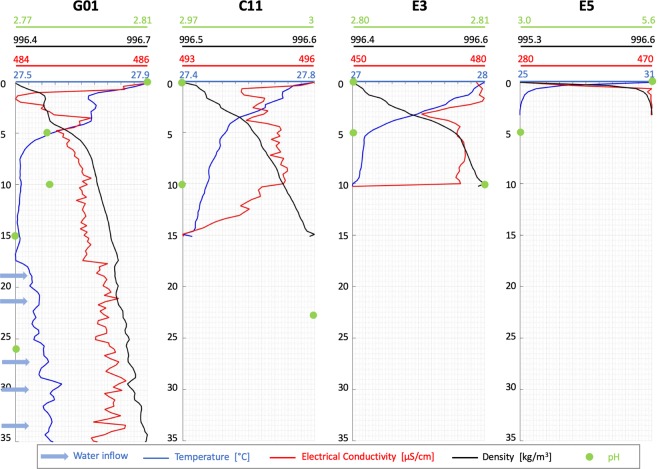
Geochemical depth profiles at selected locations in Lake Linau.

We also took geochemical measurements along a systematic grid of points with 100 m spacing across the lake. They include profiles of temperature, electrical conductivity, density and pH with depth. Here, we report measurements from the deep holes in the west (G01-35 m, C11-15 m), a profile in the middle of the lake (E3-10 m) and a location in the eastern shallow area (E5-3 m) (Fig. 7).

All temperature profiles show a strong decrease down to 5 m followed by a moderate decrease down to 15 m (Fig. 7). Below 15 m, the temperature increases. Moreover, below 15 m the temperature pattern is unstable and shows several peaks. Temperature peaks are an indication for inflow of warmer water.

Electrical conductivity generally decreases to a minimum within the top 5 m (Fig. 7). Between 5 and 15 m it slightly increases. The relatively strong decrease in C11 between 10 and 15 m can be explained by the anomalous high electrical conductivity in the top 10 m which is caused by inflow of highly saline water from hot springs. In general, the conductivity pattern is less constant than the temperature. Below 15 m, it shows parallel peaks to the temperature. This pattern cannot be explained by temperature increase only, which relates a 1 °C warming to 2% electrical conductivity increase^44^. That suggests that the warm inflowing water is also highly saline. Assuming an electrical conductivity increase caused only by temperature would lead to 60% smaller peaks.

Density constantly increases with depth at all locations. It shows a change in gradient from a weaker to a stronger increase below 5 m. The density pattern gets less constant and shows slight peaks at depth below 15 m (Fig. 7).

pH has been measured each five or ten meters over depth (Fig. 7). The most neutral pH (5.6) has been measured in the east at the lake surface and is related to river inflow near E5. The pH then decreases to the average lake pH. In the middle of the lake at E3 and at C11 pH increases with depth. This behavior is related to inflow from onshore acidic hot springs into the shallow layers of the lake at this location. The decrease in pH in the deep hole G01 over depth indicates acidic water inflow from the hole into the lake. Five different inflow zones can be located in the temperature and electrical conductivity pattern over depth (19 m, 21 m, 27 m, 30 m, 34 m) and are possibly due to fractured areas in the bedrock.

## Discussion

Our results show that combining bathymetry and geochemical measurements, in our case, is a successful approach to characterize hidden structures beneath lakes. Bathymetry uncovered deep hole structures and geochemical measurements show warm, saline, acidic fluid inflow through these holes. Our study site lies in a tectono-volcanic active environment where the deep hole structures could be volcanic vents, permeable pipe structures, pockmarks or fault intersection points.

**Figure 8:**
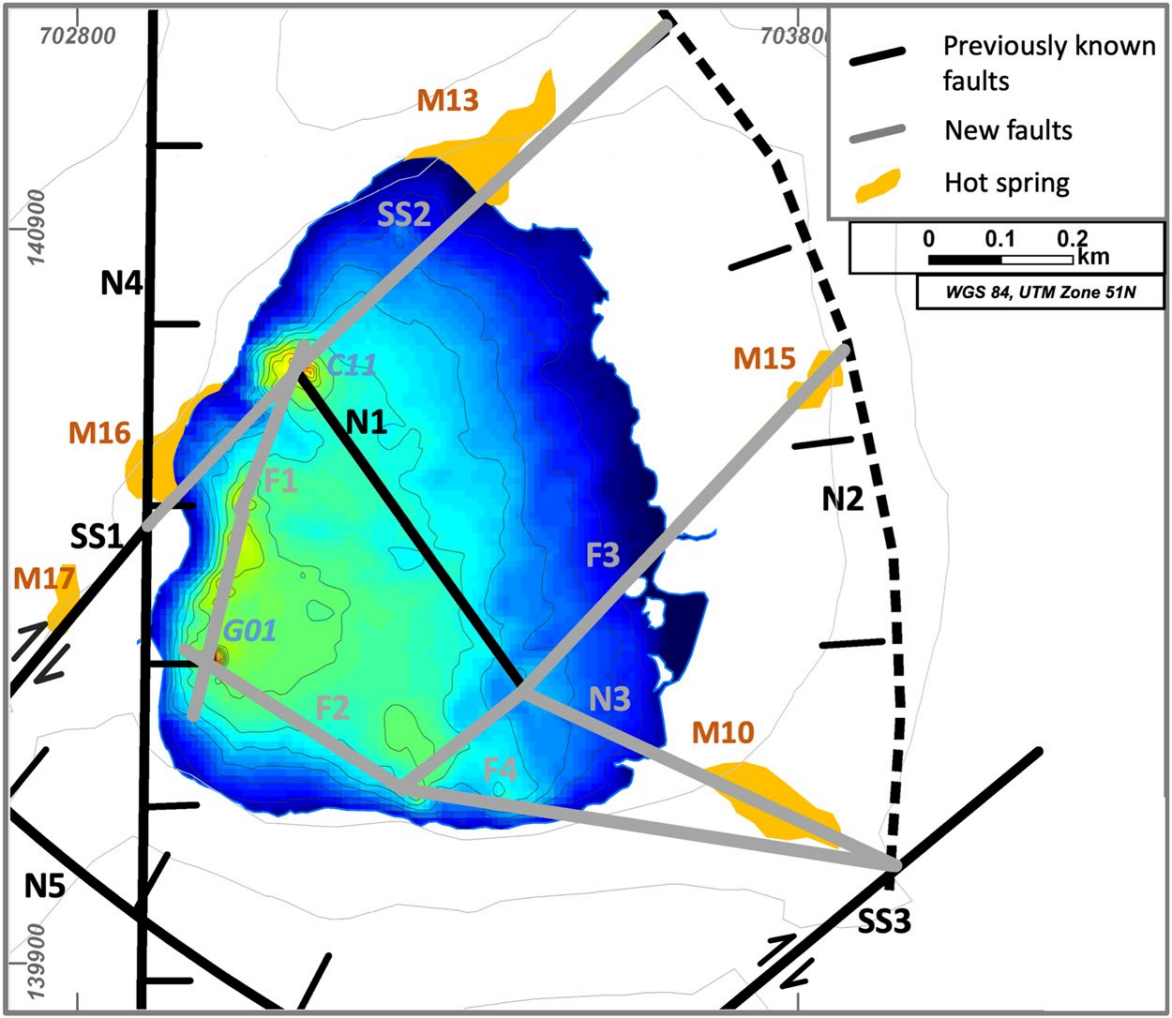
Newly discovered faults beneath Lake Linau using bathymetry in this study.

The lake bottom topography combined with outcrop data and hot spring locations shows fault traces and their intersection points. We were able to generate a detailed fault map for the Lake Linau area, where four known faults have been confirmed (SS1, N1, N2, N4) and five new faults have been discovered (SS2, N3, F1, F2, F3, F4) (Fig. 8). The 3D view shows that fault lineaments intersect where the bathymetry shows deep depressions in the lake or where hot springs occur onshore.

The most obvious lineament in the bathymetry data is connecting the deep holes in the western part of Lake Linau (F1). In the south-west a strike-slip fault (SS1) connects two hot springs onshore (M16, M17). This strike-slip fault continues towards north-east (SS2) and intersects with a NW-SE trending normal fault (N1) at the northern deep hole (C11). The strike-slip fault (SS2) builds a connection between M16, M13 and the deep hole C11 and meets with the northern border of the extension basin (N2). An extension of the normal fault N1 (i.e. N3) merges with a newly discovered fault in the south (F4) and the normal fault N2. At the deepest hole of Lake Linau (G01) a newly discovered fault F2 intersects with the normal fault N4 striking N-S. Another previously unknown fault (F3) strikes SW-NE from a deeper point in the south of Lake Linau towards a hot spring east of Lake Linau (M15), where it meets the normal fault N2 defining the northern border of an extension basin (Fig. 8).

**Figure 9:**
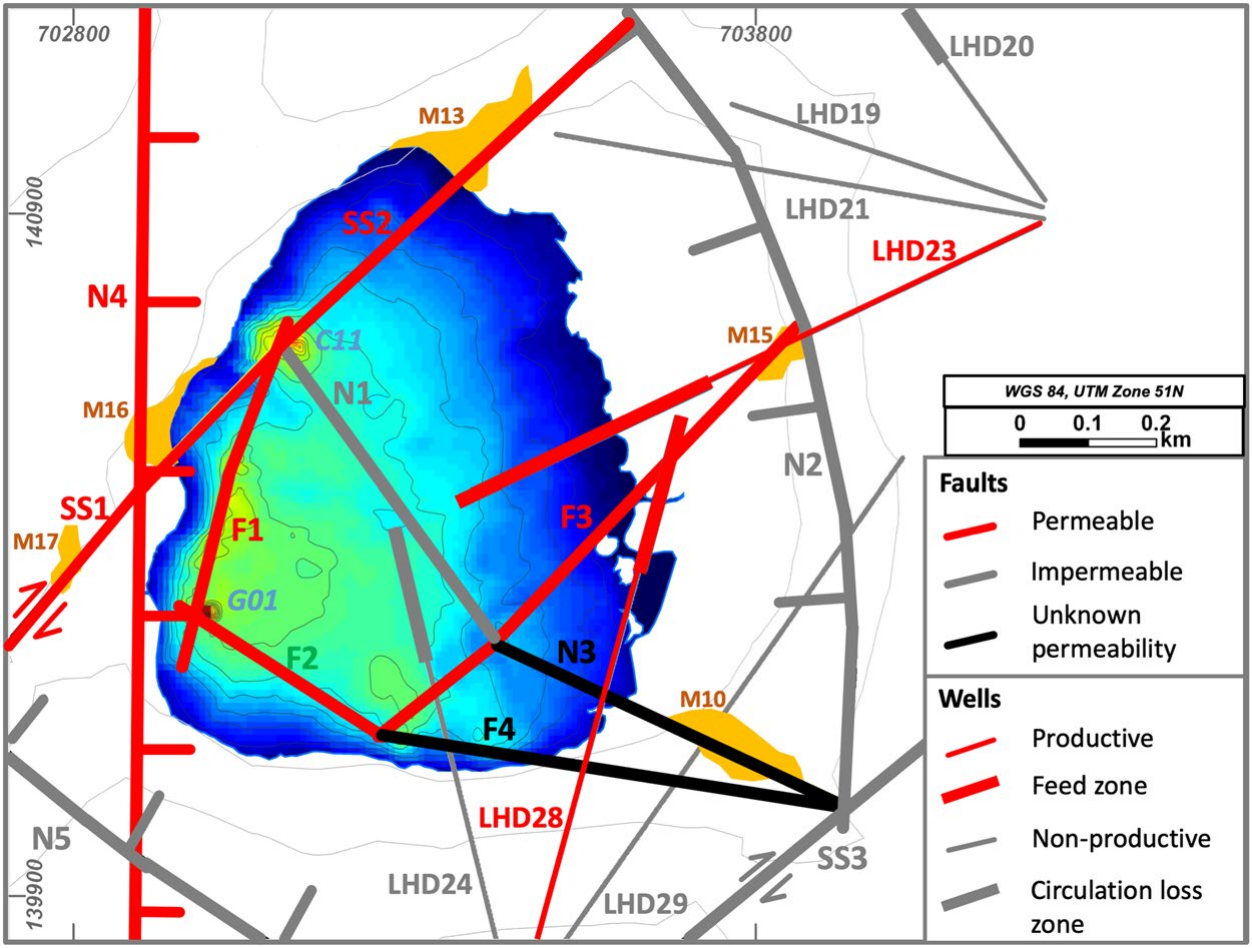
Fault permeability pattern in the Lake Linau area.

Combining this fault dataset with geochemical profiles in the lake, hot spring locations onshore and production data from wells we were able to distinguish permeable and impermeable sections of faults. Highly productive wells and hot springs near faults as well as inflow at deep holes into the lake and fault intersections in general are indicators for high permeability. In Fig. 9 red faults define highly permeable areas, while grey shows impermeable fault compartments.

Well LHD 23 and LHD 28 are located in a highly permeable reservoir compartment near F3 and show high productivities of 20 MW_e_. LHD 28 even hits the permeable fault zone F3 at 1850 m depth. Another highly productive area for wells, not targeted yet, is expected near to the deep hole G01. SS1 and SS2 are assumed to be permeable because of hot spring discharge along the faults onshore and fluid inflow into the lake from the deep hole C11. Especially at the intersection on SS1 and N4 excessive hot spring discharge is observed. F1 and F2 are assumed to be permeable because they connect several locations with fluid discharge at the lake bottom (between G01 and C11).

However, some fault intersections do not show fluid inflow into the lake. This can be due to missing measurements or due to certain permeability dynamics, where fluid pathways clog, e.g. by tectonic activity or rock alteration and scaling^45^. N1, N2, N5 and SS3 are labeled impermeable based on results from previous studies, where they act as geochemical and hydraulic boundary between reservoir compartments^36,46^. Their appearance is associated with non-productive wells (LHD 19, LHD 21, LHD 24, LHD 29).

Our results show that bathymetric data linked with geochemical measurements allows the development of conceptual geological 3D model of the subsurface. The model includes fault locations and permeability distribution beneath the lake. For the first time it is possible to locate permeable sections of faults and link them to geothermal reservoir characteristics using bathymetry and geochemistry. This technique is not limited to geothermal reservoir characterization but has a wide applicability for subsurface utilization, e.g. structural geological mapping or subsurface storage.

## Data Availability

Data and computer codes used to analyze the data in this study are available from the corresponding author on request.
